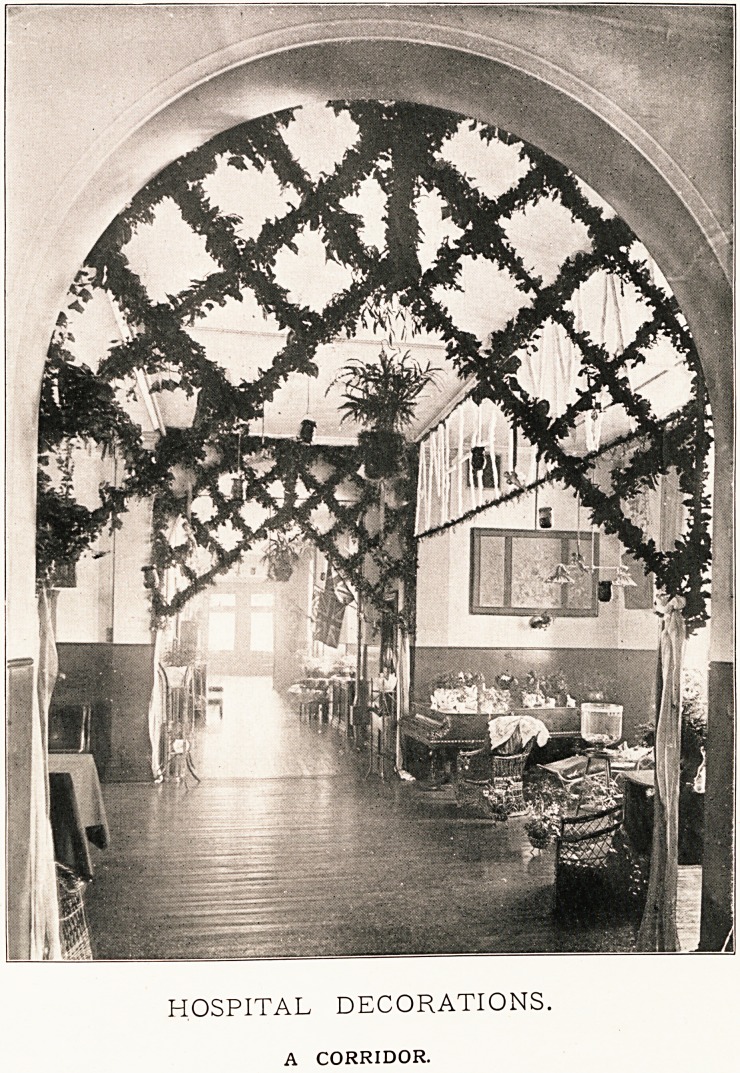# Our Christmas Appeal Portfolio

**Published:** 1900-12-15

**Authors:** 


					Supplement to "The Hospital," December 15, 1000.
OUR CHRISTMAS APPEAL PORTFOLIO.
Containing Five Hospital Illustrations:?
"WITHIN THE WARDS,"
' CHRISTMAS TREE CORNER." " DELICATE TRACERY.'
A WARD IN THE ROOF." "A CORRIDOR."
No. 742. Vol. XXIX. W
fi JP
Saturday, Dec. 15th, 1900.
Supplement to " The Hospital," December 15, 1900.
HOSPITAL DECORATIONS.
WITHIN THE WARDS.
Supplement to "The Hospital," December 15, 1900.
HOSPITAL DECORATIONS.
CHRISTMAS-TREE CORNER.
Supplement to "The Hospital," December 15, 1900
HOSPITAL DECORATIONS.
A WARD IN THE ROOF,
Supplement to "The Hospital," December 15, 1900.
HOSPITAL DECORATIONS.
DELICATE TRACERY.
Supplement to " The Hospital," December 15, 1900.
n >
HOSPITAL DECORATIONS.
A CORRIDOR.

				

## Figures and Tables

**Figure f1:**
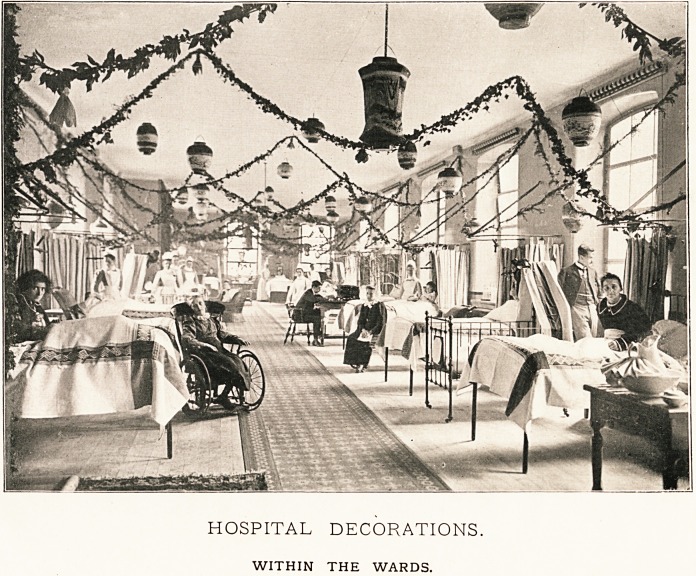


**Figure f2:**
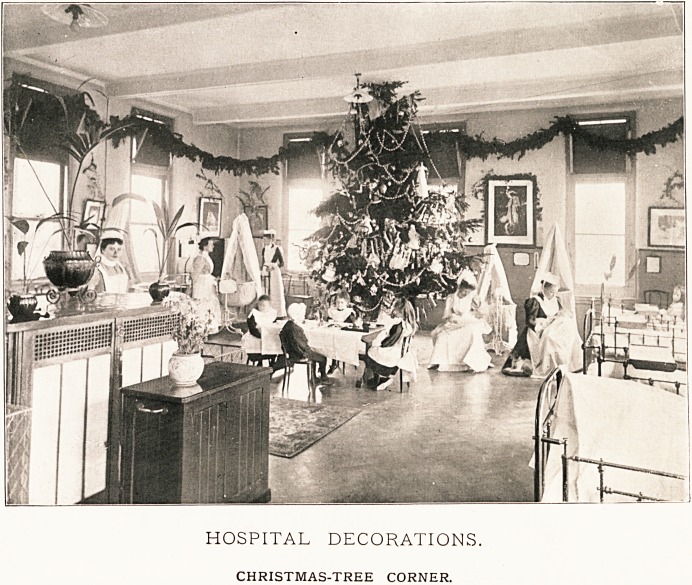


**Figure f3:**
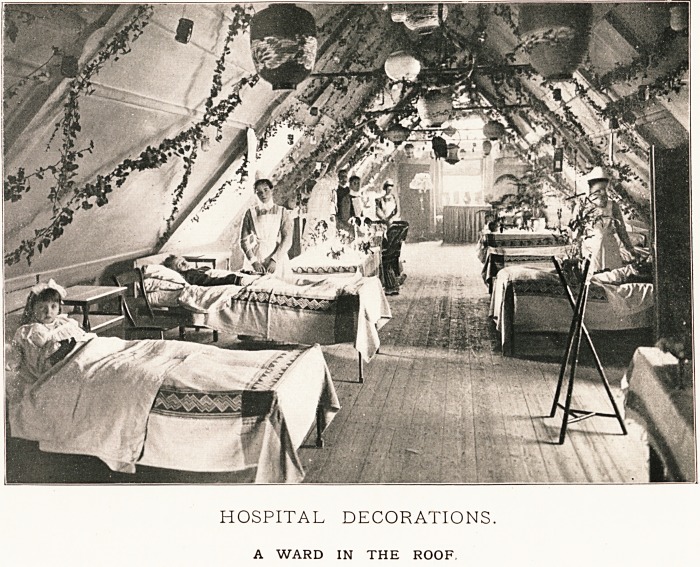


**Figure f4:**
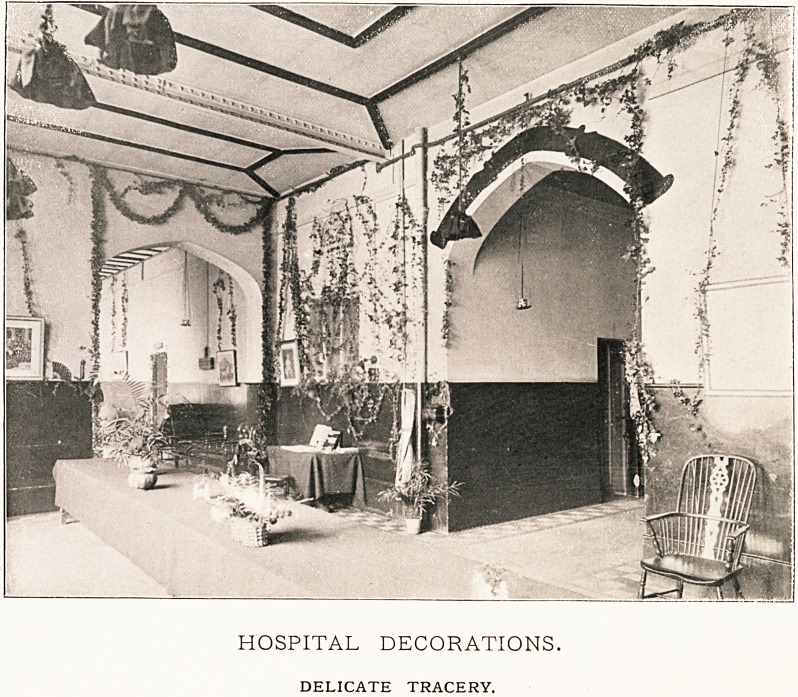


**Figure f5:**